# Farmers’ knowledge, attitudes, and practices towards the adoption of hybrid rice production in Bangladesh: an PLS-SEM approach

**DOI:** 10.1080/21645698.2022.2140678

**Published:** 2022-11-22

**Authors:** Paresh Kumar Sarma, Mohammad Jahangir Alam, Ismat Ara Begum

**Affiliations:** a Senior Scientific Officer (SG), Bangladesh Agricultural University Research System (BAURES), Bangladesh Agricultural University, Mymensingh, Bangladesh; b Professor, Department of Agribusiness and Marketing, Bangladesh Agricultural University, Mymensingh, Bangladesh; c Professor, Department of Agricultural Economics, Bangladesh Agricultural University, Mymensingh, Bangladesh

**Keywords:** Hybrid rice adoption, KAP, PLS-SEM, production, SmartPLS3 software

## Abstract

Although the government policy supports the adoption of hybrid rice in Bangladesh but there is no significant progress has been made over the last decades. The study aimed to determine farmers’ knowledge, attitudes, and practices (KAPs) of hybrid rice adoption, and the causal relationship between them in Bangladesh. Using a stratified random sampling technique, 244 cross-sectional surveys were done from the Sherpur district of Bangladesh. The descriptive statistics, two-round Delphi method, and partial least squares structural equation modeling (PLS-SEM) were used to estimate latent variables and identify the causal relationship between KAPs. The results revealed that farmers’ knowledge and attitudes were directly and significantly associated with the adoption of hybrid rice. This implies that farmers who are well informed about the usage of hybrid rice and are likely to embrace it and raise their revenue, can be influenced by knowledge. The study found a significant positive role for knowledge about hybrid rice, attitudes, and practices in adopting hybrid rice. This study provides a primary understanding of the relationship between psychological factors and the adoption of hybrid rice technology to increase rice farm productivity. The findings suggest that policymakers, researchers and development agencies should focus on farmers’ psychological factors in improving the adoption of hybrid rice in Bangladesh.

## 1 Introduction

Bangladesh is primarily an agrarian economy with a dense population, and food security remains a major concern. Agriculture is vital to the creation of rural employment and the generation of income in Bangladesh and is thus regarded as the country’s lifeline. Rice is the primary diet of half the world’s population and 70% of South Asian countries^1–3^ and it is the primary income source for one-fifth of the total world’s population.^4,5^ Bangladesh is the third global rice-producing country^6^ and 70% of the calorie come from rice. Researchers have been putting lot of efforts to increase rice production to meet food security for growing populations in many developing countries. In this context, due to the use of agricultural land for industrialization, land fragmentation and urbanization, agricultural land is shrinking day by day. In spite of land expansion is not possible, the food production system will need to be more productive to reduce poverty and attain food security for all. Thus, new advanced technologies are needed to improve productivity while maintaining sustainable farmers’ profitability with a minimum negative impact on the environment.^7–9^ Among the advanced technologies, the hybrid rice is the potential alternative technology to increase rice productivity and farmers’ income to ensure food security with fix amount of land.

In Bangladesh, hybrid rice had been introduced in 1996 but the adoption of hybrid rice is stagnant in Bangladesh. The creation and dissemination of improved rice varieties is the most prominent of all the different attempts to boost rice yield. The yield and the quality of the crop are greatly influenced by the quality of the sowed land since they are the most crucial input parameters in agriculture.^8^ Due to a lack of information flow and expertise with the new kinds after the release of hybrid types in 1999, acceptance was gradual. Following the green revolution, there was a renewed interest in improving rice production due to the innovation of hybrid rice and the success of some nations, like China, in achieving rice food security by promoting hybrid rice adoption.^10^ This is mainly because hybrid rice provides a yield gain of 15–20% over the conventional rice varieties.^11–13^ Because it is crucial to cover large regions with rice cultivation to maintain food security, the Bangladeshi government has implemented a number of initiatives and courses of action to encourage the wider use of hybrid rice. By enhancing irrigation capabilities and implementing contemporary high-yielding cultivars in the Boro, dry rice season, Bangladesh is one of the best countries at increasing rice output.^14^ However, in Bangladesh, the use of hybrid rice is primarily restricted to the Boro rice season.

**Figure 1.**
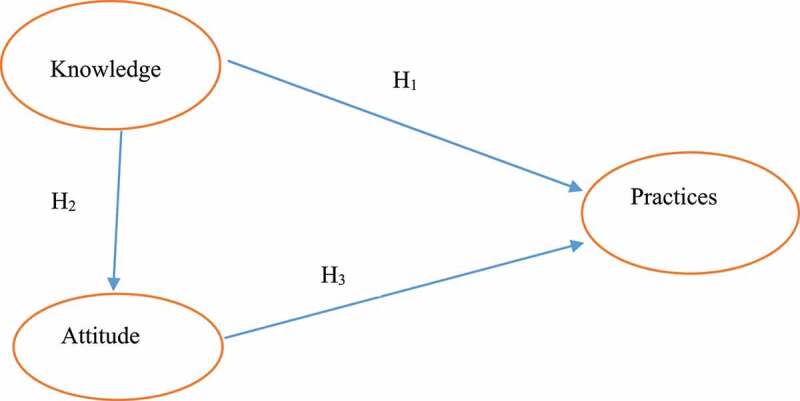
Conceptual framework.

Very few studies^1,5,15–18^ have been conducted to examine the factors determining the adoption of hybrid rice in Bangladesh. Most of the previous studies were conducted based on Rogers’ adoption of innovation theory but no study on psychological farmers’ behavior theory. Considering these issues in mind, the study has examined farmers behavior regarding knowledge attitudes and practices (KAP) toward adoption of hybrid rice. .The KAP study by Blooms (1956) and empirical research on the acceptance of innovation in agriculture served as the foundation for this study’s framework, which is depicted in Figure 1. The conceptual framework described how farmers’ knowledge, attitudes, and practices all have a direct impact on whether hybrid rice varieties are adopted. The findings of this study will help researchers in the field of agricultural technology adoption in gaining a deeper understanding on what factors influence hybrid rice producers’ attitudes toward production and how attitudes are formed.

**Figure 2.**
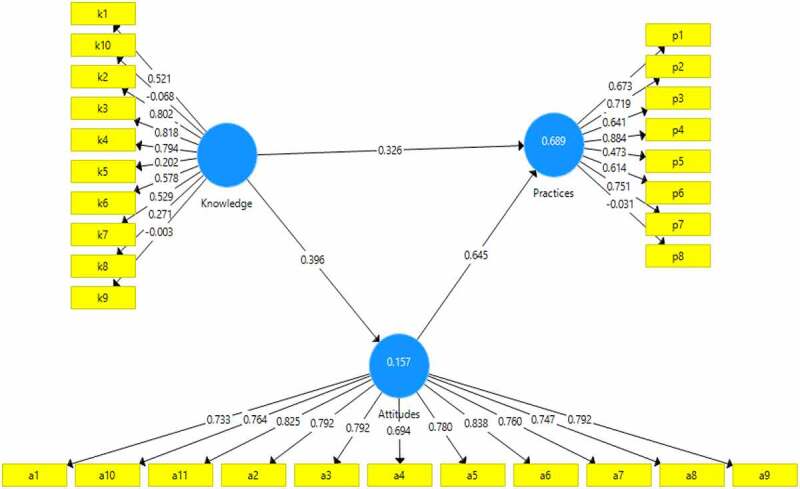
The PLS-SEM modeling results.

The paper intends to test the following research hypotheses:

• *H_1_*: Farmer’s knowledge regarding the hybrid rice has been directed association with hybrid rice practices

• H*_2_*: Farmer’s knowledge has an association with farmer’s attitude to adopt hybrid rice practices.

• *H_3_*: Farmer’s attitude has a direct association with hybrid rice adoption in the study area.

This paper is organized as follows. After the introduction in Section 1, materials and methods are presented in Section 2. The results of this study was presented in the section 3 discussion in section 4 and finally conclusion and recommendation in the section 5.

## 2 Materials and Methods

### 2.1 Area of the Study

The study was carried out in Nakla Upazila of Sherpur district in Bangladesh from in the year of 2022, which was purposefully chosen due to the high degree of rice production. Nakla Upazila has an area of 174.80 square kilometers and is located between 24°53’ and 25°02’ north latitudes and 90°07’ and 90°15’ east longitudes. It is surrounded in the north by Nalitabari Upazila, in the east by Phulpur and Haluaghat Upazilas, and in the west by Sherpur Sadar Upazila.

### 2.2 Data Collection

Mainly primary data were used in this research. Before conducting the survey, the two rounds of Delphi techniques were used to determine the indicators against each latent variable. After receiving the expert opinion regarding the indicators, the previous studies were consulted to finalize the indicators. Then survey questions related to the indicators were developed. Most of the questions were closed-ended. From January to March 2022, primary data were collected by face-to-face interviews with farmers using pre-tested structured questionnaire. The data were collected by nine trained enumerators who read the questions to the farmers and recorded their replies on tablet computers. Primary data on farmers’ socioeconomic and demographic factors, farmers’ knowledge, attitudes and practices, rice varieties cultivated, and other farm features were obtained.

### 2.3 Sampling Technique

A stratified random sampling technique was used for the study. The Department of Agricultural Extension (DAE0, helped to make a list of all the villages. Then, the villages where rice is cultivated and villages where rice is not cultivated were separated, and the researcher considered the villages where rice is cultivated. All remaining villages were then divided into three homogenous groups (strata) based on village accessibility. Villages are classified into three types: “villages with good accessibility all year,” “villages with low accessibility,” and “villages with highly restricted accessibility.” In this situation, accessibility is determined by both road conditions and distance from a city. Villages with extremely restricted access were not taken into consideration because of the high intensity of the fieldwork. Based on the crops that predominated, each of the two remaining village groupings was splitted into two classes (rice in the target ecology as the major crop and rice in the target ecology as the minor crop). Four strata of villages were created using the “accessibility” and “dominant crop” criteria: villages with good access but rice as the major crop, villages with good access but rice as the minor crop, villages with good access but rice as the major crop, and villages with poor access but rice as the minor crop. Eight villages were chosen at random from each stratum, for a total of 32 villages out of 117. Then, eight households were randomly chosen from each of the 32 villages, for a total of 256 rice farming households. However, only 244 households’ worth of data could be used for the study because 12 households didn’t participant for the study due to time constraint. Based on their adoption status, these families were then classified as adopters or nonadopters. If a farm household cultivates a certain hybrid rice in at least one of its rice plots, it is considered to be an adopter of that hybrid rice.

### 2.4 Data Analytical Procedure

Descriptive and inferential statistics, as well as KAP modeling, were used to analyze the data. The partial least squares structural equation modeling (PLS-SEM) analysis with SmartPLS3 software was used to analyze the measurement and find the causal relationship between KAPs and hybrid rice adoption.

#### 2.4.1 SmartPLS Analysis

The PLS-SEM statistical approach was used to estimate cause-and-effect connection models utilizing latent variables.^5^ To examine the structural effect of KAPs on hybrid rice uptake, multiple regression and PLS-SEM are appropriate choices. We utilized cross-sectional data and Smart PLS statistical tools to determine the direct and indirect impacts of KAP on hybrid rice adoption. The study employs SEM as its standard reporting methodology. SEM is a second-generation method for multivariate data analysis that evaluates additive and linear causal models that are theoretically maintained.^19,20^ Many published studies in the management and marketing fields make extensive use of PLS-SEM.^21^ The study has employed a PLS-SEM model because our sample is rather small and we are unsure about the distribution of the data acquired through surveys. For PLS-SEM modeling, a two-step process is used, with the first measurement being performed on the model to confirm the model’s validity and dependability of study components.^20^ The second stage entails using structural model linkages and bootstrapping significance thresholds.^20^ STATA-16 was employed to conduct a demographic statistical analysis, and then SmartPLS 3.3 software was used to estimate a structural equation model.

#### 2.4.2 Construction of Latent Variable KAPs

Farmers’ knowledge, attitudes, and practices toward hybrid rice adoption were measured using a total of 29 observations, including 10 observations for knowledge, 11 observations for attitudes, and 8 observations for practices, based on two rounds of Delphi method application (expert opinion) following Nyi et al.^22^

**Knowledge**: In the knowledge area, respondents respond yes/no to 10 questions about the manner of accessing knowledge. The binary scores for each question were added together to give a knowledge score ranging from 0 to 10. The answer “yes” was coded as 1, while the answer “no” was coded as 0. A total score of 0–10 with an overall score of greater than indicates more favorable knowledge of the hybrid rice adoption. On the other hand, a knowledge index was created from the raw knowledge scores of each respondent using the formula given by ^22,23^].

$$Index\;of\;knowledge=\frac{\begin{array}{c} Number\;of\;\\ correct\;responses \end{array}}{\begin{array}{c} Total\;number\;of\\ \;knowledge\;observations \end{array}}$$

**Attitude**: The attitudes section consisted of 11 observations regarding the positive attitudes toward hybrid rice adoption. We used a five-point Likert scale. Each statement was scored with a 5 for “strongly agree,” a 4 for “agree,” a 3 for “neutral,” a 2 for “disagree,” and a 1 for “strongly disagree.” The attitude ratings were added together to obtain an overall attitude score ranging from 0 to 95 points. For attitude scores, the Cronbach’s alpha coefficient ranged from 0.67 to 0.70, showing a satisfactory level of internal consistency.^4,18^

The attitude index was then created using the following Nyi et al.,^22,23^ taking the total of the respondents’ actual scores.

$$Attitude\;index=\frac{\begin{array}{c} Sum\;of\;scores-Minimum\;\\ possible\;scoreses \end{array}}{\begin{array}{c} Differences\;between\;maximum\;\\ and\;minimum\;possible\;scores \end{array}}$$

**Practices**: There were 8 items on the questionnaire that assessed the practices of adopting hybrid rice varieties. We used a five-point *Likert* scale. Each statement was scored with 5 for “strongly agree,” 4 for “agree,” 3 for “neutral,” 2 for “disagree,” and 1 for “strongly disagree.” Cronbach’s alpha coefficient of the “practices” to adopt rice varieties varied from 0.70 to 0.74, indicating a satisfactory level of internal consistency.^4,18^ We calculated the mean percentage score by dividing each of the KAP mean scores by their greatest potential score and multiplying them by 100. On the other hand, the actual scores of the respondents were added together, and the attitude index was determined using the method below.^22^

$$Practices\;index=\frac{\begin{array}{c} Sum\;of\;scores-Minimum\;\\ possible\;scoreses \end{array}}{\begin{array}{c} Differences\;between\;maximum\;\\ and\;minimum\;possible\;scores \end{array}}$$

**Cross-loadings**: Every construct piece scored higher on its own construct than any other. Consequently, the constructs’ discriminant validity was sufficient (Table 1). Hair et al. (2014) argued that factor loadings should be more than 0.50. Table 1 illustrates the factor loadings’ outcomes. Out of the 29 factors, the loading values for approximately 19 were less than 0.50. The range of the factor loadings was −0.088 to 0.903. An accurate estimation of the reliability for a particular test may be obtained using the test reliability approach known as Cronbach’s alpha with just one delivery of the test. When divided into two half tests, all potential item combinations would result in an average reliability coefficient known as Cronbach’s alpha. Cronbach’s alpha reliability coefficient normally ranges between 0 and 1. George and Mallery^38^ provide the following rules of thumb: excellent = α ≥ 0.90, good = α ≥ 0.80, acceptable α ≥ 0.70, questionable α ≥ 0.60, poor α ≥ 0.50 and unacceptable α ≤ 0.50. We find that all the estimated Cronbach’s alpha values are greater than 0.50. The estimated Cronbach’s alpha range is 0.645 to 0.943. This means that Cronbach’s alpha indicates good internal consistency of the items in the scale, which does not mean that the scale is unidimensional. The overall Cronbach’s alpha value also noted that an alpha of 0.786 was probably a reasonable goal. Multicollinearity occurs when two or more predictors in the model are correlated and provides redundant information about the response. Multicollinearity was measured by variance inflation factors (VIFs) and tolerance. If the VIF value exceeds 4.0 or the tolerance is less than 0.2, then there is a problem with multicollinearity^39^, but some papers argue that a VIF<10 is acceptable^40^. In most cases, a rule of thumb commonly used in practice is that if a VIF is >10, there is high multicollinearity. The range of estimated VIF values was 1.031 to 4.596 (Table 1). This means that all VIF values were acceptable, with no multicollinearity among the indicators.

**Table 1.** KAPs of hybrid rice adaptation farmers.

KAP	Items	Question statements	Mean	S. D	Factors Loading	VIF	*Cronbach’s* alpha
Hybrid rice s knowledge	K_1_	Do you know the differences between (for sowing) and grains (for eating)?	0.402	0.492	0.285	1.673	0.645
	K_2_	Do you head about the hybrid rice?	0.213	0.411	0.850	1.906	
	K_3_	Do you know subsidized hybrid rice s are available in local market?	0.262	0.442	0.903	2.516	
	K_4_	Do you know that hybrid s has good germination than that of grains?	0.303	0.462	0.812	2.462	
	K_5_	Do you know that hybrid s has higher genetic purity than that of grains?	0.197	0.399	0.169	1.163	
	K_6_	Is there lower rate needed by using hybrid s?	0.352	0.480	0.452	1.902	
	K_7_	Do you know that hybrid s can be used as s for next time/seasons?	0.607	0.491	0.386	1.911	
	K_8_	Do you know that using hybrid s can increase the rice yield?	0.344	0.477	0.212	1.720	
	K_9_	Do you know that the label card color is included in the bag of hybrid s?	0.770	0.422	0.054	1.076	
	K_10_	Do you know hybrid rice s resistant to pests and diseases, lodging, and tolerant to abiotic stresses	0.164	0.372	−0.088	1.046	
Hybrid rice s attitude	A_1_	The paddy yield can increase approximately 10%-20% by using hybrid rice s.	1.828	0.888	0.593	2.155	0.943
	A_2_	Hybrid rice s can protect from the borne diseases.	2.057	0.893	0.551	2.776	
	A_3_	Quality s can reduce the amount of rate per acre.	2.008	0.848	0.460	3.308	
	A_4_	By using certifies s, it is easier to grow and manage the plant.	2.328	0.886	0.666	2.983	
	A_5_	The practice of mixing with other varieties should be minimized by using hybrid s.	1.836	0.856	0.521	3.405	
	A_6_	Hybrid s have a better response to use inputs.	2.115	1.069	0.565	4.531	
	A_7_	Hybrid s give a good percentage of germination.	2.148	1.065	0.678	2.880	
	A_8_	The practice of mixing with weed s should be minimized by using hybrid s.	2.385	0.913	0.810	2.653	
	A_9_	The use of hybrid s can easily be harvested due to uniform ripening.	2.410	1.170	0.786	4.300	
	A_10_	With hybrid cultivation, the crop has good quality and good market competitiveness.	2.000	1.060	0.775	3.436	
	A_11_	Hybrid rice is shorter field duration than local varieties	2.107	0.889	0.811	4.596	
Hybrid rice s practices	P_1_	I have used hybrid rice s for better yield and high weight of rough rice	1.475	0.592	0.674	1.968	0.771
	P_2_	I always used hybrid rice s for less requirement and ensuring germination	1.689	0.681	0.751	2.209	
	P_3_	Spectacular and eye-catching views of rice field	1.664	0.734	0.673	2.117	
	P_4_	Hybrid rice s is suitable for low lying areas production	1.607	0.611	0.882	3.092	
	P_5_	Hybrid rice are easy to postharvest operation, market demand and high price in the market.	2.262	0.736	0.482	1.492	
	P_6_	Hybrid rice fetches a good price in the local rice market	1.844	0.704	0.596	1.871	
	P_7_	Hybrid rice s are of good quality, available in the market through government/cooperatives/BADC	1.467	0.658	0.109	1.836	
	P_8_	Hybrid rice are more productivity than nonhybrid rice production.	3.689	0.669	−0.055	1.031	

## 3. Results and Discussion

### 3.1 Socioeconomic Characteristics

The Table 2 shows that the socioeconomic factors have several implications for the adoption of new agricultural technologies and practices. We found that the majority of sample farmers were male. Among the total sample farmers, 90.16% of adopters and 51.64% of non-adopters were male, and 9.84% of adopters and 25.41% of non-adopters were female. The majority of adopters (85.25%) and nonadopters (72.13%) were married, while only 1.64% of the adopters were divorcees and 6.56% of nonadopters were widows, suggesting that greater proportions of hybrid rice farmers in the study area were married. About 57% of the adopters was in the age between 36–45 years, 28.69% between 46–55 years of age of the adopter’s farmers. On the other hand, about 45.90% farmer’s age was between 36–45 years and 31.97% between 46–55 years in the non-adopter farmers group. Approximately 57.38% of the farmers were within the age bracket of 36–45 years, and only 1.64% were above 65 years of age. However, 45.90% of the non-adopters age were between 36–45 years and they are statistically significant with the t-value of −5.066. Most of farmers were young who are easily adopt new technology because old farmer are depicting the risk-averse nature of the older and aged farmers, who are usually more conservative than the younger ones. Income places farmers in a good financial position to buy all the necessary inputs and adopt hybrid rice technologies.

**Table 2.** Socioeconomic characteristics of survey respondents.

Variables		Adopters (N = 122)		Non-adopters (N = 122)		Total (N = 244)		t-test
		Frequency	%	Frequency	%	Frequency	%	
Sex	Male	110	90.16	63	51.64	173	70.90	−4.0106***
	Female	12	9.84	31	25.41	43	17.62	
Marital status	Single	11	9.02	23	18.85	34	13.93	0.5525
	Married	104	85.25	88	72.13	192	78.69	
	Divorced	2	1.64	3	2.46	5	2.05	
	Widowed	5	4.10	8	6.56	13	5.33	
Age (years)	18–35	12	9.84	5	4.10	17	6.97	−5.06***
	36–45	70	57.38	56	45.90	126	51.64	
	46–55	35	28.69	39	31.97	74	30.33	
	56–65	3	2.46	11	9.02	14	5.74	
	65 and above	2	1.64	11	9.02	13	5.33	
Monthly income	Less than 30000	5	4.10	33	27.05	38	15.57	4.35***
	20001–50000	80	65.57	71	58.20	151	61.89	
	50001–70000	22	18.03	13	10.66	35	14.34	
	70001–100000	6	4.92	3	2.46	9	3.69	
	100001 and above	9	7.38	2	1.64	11	4.51	
Family Size	Less than 5	13	10.66	4	3.28	17	6.97	0.92
	6 to 10	43	35.25	48	39.34	91	37.30	
	11 to 15	60	49.18	56	45.90	116	47.54	
	16 and above	6	4.92	14	11.48	20	8.20	
Farming Experience	Less than 5 years	3	2.46	8	6.56	11	4.51	6.12***
	6–10 years	33	27.05	35	28.69	68	27.87	
	11–15 years	73	59.84	41	33.61	114	46.72	
	16 and above	13	10.66	38	31.15	51	20.90	
Education level	Primary education	9	7.38	47	38.52	56	22.95	3.45***
	Secondary education	67	54.92	49	40.16	116	47.54	
	Graduate education	35	28.69	19	15.57	54	22.13	
	Post-graduate education	11	9.02	7	5.74	18	7.38	
Land holding	Own land	61	50.00	69	56.56	130	53.28	2.89**
	Rented Land	23	18.85	11	9.02	34	13.93	
	Borrowed Land	5	4.10	5	4.10	10	4.10	
	Contracted land	5	4.10	7	5.74	12	4.92	
	Share cropped	6	4.92	5	4.10	11	4.51	
Membership of FBO	Yes	79	64.75	65	53.28	144	59.02	−0.43
	No	43	35.25	57	46.72	100	40.98	
Hybrid rice seed availability	Yes	103	84.43	78	63.93	181	74.18	−4.77***
	No	19	15.57	44	36.07	63	25.82	

Monthly farm income refers to the total amount of money earned by a rice farmer from rice production. The respondents were asked to estimate the total amount of income they earned from rice production in BDT per month. This was used as their monthly income. A high proportion (65.57%) of the adopter earned about BDT 20001–50000, while only (4.10%) of the adopters earned about less than BDT 30000, whereas 27.05% of non-adopters earned less than BTT 30000 this is followed by 18.03% who had BDT 50001–70000 monthly income, 4.92% had BDT 70001–100000 monthly income, and 7.38% had 100001 and above, while 10.66%, 2.46% and 1.64% of non-adoption farmers, respectively. The mean comparison t-test value of two groups is 4.357. It means that the significant difference between the two groups prevails.

We found that 35.25% of the adopters had a family size of 6–10 persons, while 4.92% had 16 and above. On the other hand, approximately 39.34% of the non-adopters had 6–10 members per household, and 3.28% had fewer than 5 members per household. This suggests that non-adopters had a larger family size than adopters in the study area. The findings also revealed that the majority of adopters (64.75%) and non-adopters (53.28%) were engaged in membership amounts in the farmer-based organization, and 84.43% of rice farmers easily exceeded the hybrid rice seeds of adopters, whereas only 63.93% of farmers had the option to access hybrid rice production in the study area. The t-test estimate value was (−2.770) indicate the difference between two group.

### 3.2 Descriptive Statistics of Latent Variables

#### Knowledge Index

Farmers’ knowledge was assessed using a knowledge index. Following the determination of each farmer’s knowledge score, they were divided into three groups using minimum and maximum scores as the points of differentiation. Table 3 revealed that a high degree of knowledge regarding hybrid rice was held by 30.33% of adopters, followed by a medium level by 55.74% and a low level by 13.93%. Conversely, 8.20% of non-adopters had a high degree of understanding regarding hybrid rice, while the percentages with medium and low levels of knowledge were 25, 41, and 66.39%, respectively. The average knowledge index value of the two groups differed significantly at the 1% level, according to the results of the t-test. These findings are consistent with those of Nazuri et al.,,^6^ who found that knowledge is a valuable asset and a driving force behind the adoption of hybrid rice.

**Table 3.** Knowledge index of sample farmers.

Knowledge Index	Adopter (n = 122)		Nonadopter (n = 122)		t-test	Sig.
	No	Percentage	No	Percentage		
Low (0.20)	17	13.93	81	66.39	4.627***	00.00
Medium (0.20–0.94)	68	55.74	31	25.41		
High (>0.94)	37	30.33	10	8.20		
**Total**	**122**	**100.00**	**122**	**100.00**		

Notes: *** is significant at the 1% level. Values in parentheses indicate percentages.

Table 3 below displays the respondents’ knowledge of the enhanced adoption of hybrid rice. The results demonstrate that most farmers had a high degree of knowledge, with a frequency of 405, or 91.6%, and a mean score of 4.30. The outcome also showed that a moderate amount was reported by the second group of respondents, accounting for 33 frequency counts or 7.5% of respondents. As calculated in Table 1 below, the remaining respondents had a poor degree of expertise, with a frequency of 4 representing 0.91% of them.

#### Attitude Index

The degree of a psychological object’s positive or negative influence is referred to as attitude. Regarding the farmers’ attitudes, Table 4 shows that 33.82% of the adopters’ sample farmers had a positive attitude toward the production of hybrid rice. However, 57.35% and 8.82% of the farmers who adopted hybrid rice farming had average and poor views, respectively. In contrast, 18.46% of the farmers in the nonadopters’ sample had a high attitude toward hybrid rice, compared to 49.23% and 32.316% of the farmers in the non-adopter’s sample who had a medium attitude and a low attitude, respectively. The t-test resulted in highly significant differences between the two groups’ average attitude index at the 1% level.

**Table 4.** Attitudes toward hybrid rice.

Attitude Index	Adopter (n = 122)		Nonadopter (n = 122)		t test	Sig.
	No	Percent	No	Percent		
Low (<0.28)	11	8.82	39	32.31	3.398***	0.000
Medium (0.28–0.93)	70	57.35	60	49.23		
High (>0.93)	41	33.82	23	18.46		
**Total**	**122**	**100.00**	**122**	**100.00**		

Notes: *** is significant at the 1% level. The value in parentheses indicates the percentage.

#### Practices Index

The findings on farmers’ degree of experience with the upgraded hybrid rice technology are displayed in Table 5 below. The results showed that most respondents reported a high level of practices, with 235 frequency counts, or 53.2 respondents, and a mean score of 3.71 on the degree of practice. The majority of respondents (42.5%) came into the category of intermediate-level practitioners, with a frequency count of 188, while the minority (4.3%) fell into the category of low-level practitioners on the enhanced hybrid rice.

**Table 5.** Practices toward hybrid rice.

Practices Index	Adopter (n = 122)		Nonadopter (n = 122)		t test	Sig.
	No	Percent	No	Percent		
Low (< 0.28)	14	11.48	39	31.97	3.398***	0.031
Medium (0.28–0.93)	63	51.64	58	47.54		
High (>0.93)	45	36.89	25	20.49		
**Total**	**122**	**100.00**	**122**	**100.00**		

Note: *** is significant at the 1% level. Values in parentheses indicate percentages.

### 3.3 Association between KAPs of Survey Samples

The association among Knowledge, Attitude, and Practice scores has a strong relationship (Table 6). The findings showed that an increase in knowledge score was substantially associated with an increase in positive attitude, but there was no association with practice. Farmers’ practices in Bangladesh were shown to be influenced by an increase in positive attitudes.

**Table 6.** Pearson correlation.

	Knowledge	Attitude	Practices
Knowledge	0.095*		
Attitude		0.056	
Practices			0.327**

The correlational analysis demonstrated that all independent components, knowledge and practice, had a positive correlation with adoption at 1% level of probability (Table 6). As a result, the independent variables knowledge and practice had modest correlations. We find that the factors’ relationships with adoption were (r = 0.469, p = .000) for knowledge and (r-0.578, p = .000) for practice The Pearson chi-square test revealed a positive and significant link between knowledge (χ2 = 41.49; p = .001) and adoption expertise; adopters outperformed nonadopters (more than twofold). The chi-square test confirmed this conclusion, revealing that farmers had positive feelings not only for the region in issue, but also for the extension agents that presented the technology to the farmer.

### 3.4 PLS-SEM Results

The PLS-SEM was applied to check the extant causal relationships among the farmers’ psychological factors based on farmers’ behavior theory. The psychological factors were constructed based on different observations such as knowledge, attitude and practices. To determine whether or not the proposed hypotheses were sound, we first look at the known linkages of cause and effect between the various variables. The PLS-SEM approach was used to examine the effect of farmers’ knowledge on hybrid rice, farmers’ adaption strategies against the farmers’ psychological factors (See Figure 2) and the SmartPLS structural equation modeling results (Table 7).

**Table 7.** Total effect.

Hypothesis path	Original Sample (O)	Sample Mean (M)	Standard Deviation (STDEV)	T Statistics (|O/STDEV|)	P-Values	Decision
Attitude -> Practices	0.501	0.499	0.052	9.612	0.000	Supported
Knowledge -> Attitude	0.469	0.496	0.048	9.800	0.000	Supported
Knowledge -> Practices	0.724	0.733	0.040	17.963	0.000	Supported

It was revealed that various items with their outer loadings under each indicator were highly significant. Based on the model framework and this research hypothesis, the structural equation model can be seen in Figure 4. Standard bootstrapping was used with 244 sample observations to examine the significance of path coefficients.^6^ *H_1_* shows that knowledge has positive and significant effects on hybrid rice adoption (practices) (β = 0.326, t = 17.963, p < .05). *H_2_* shows significantly positive effects of knowledge on attitude ((β = 0.396, t = 9.800, p < .05). Similarly, *H_3_* shows positive and significant effects of attitude on hybrid rice adoption (β = 0.645, t = 9.612, p < .05). The path coefficients of the factors concerned with hybrid rice adoption of rice farmers show that knowledge (0.724) is the important factor of hybrid rice adoption followed by attitudes (0.501) and practices (0.469) in Table 7.

#### 3.4.1 Validity and Reliability Test

Convergent validity is an assessment based on the correlation between the reflexive indicator and latent variable scores. According to Chin^41^, loading factors ranging from 0.6 to 0.7 can be tolerated as long as the indication is not the only indicator in the latent variable. The results revealed that all factor-loading values for the majority of the indicators were more than 0.5. This signifies that all indicators are legitimate and usable since they fulfill the convergent validity criterion. The Cronbach’s alpha value and the dependability value of each contract may be used to do reliability testing. If the composite reliability value is 0.70 and the Cronbach’s alpha value is more than 0.7, the variable is considered to have good reliability. According to the findings (Table 8), which indicated that all constructs tested in this study had Cronbach’s alpha values of 0.7 and composite reliability > 0.70, all constructs were either trustworthy or all indicators accurately represented the constructs that were developed.

**Table 8.** Validity and reliability measurement.

	Cronbach’s Alpha	Dijkstra-Henseler’s (rho_A)	Composite Reliability	Average Variance Extracted (AVE)
Attitudes	0.934	0.939	0.943	0.601
Knowledge	0.686	0.809	0.737	0.495
Practices	0.757	0.825	0.827	0.516

Internal consistency is less accurate than composite dependability. It may be used in conjunction with PLS-SEM to accommodate various loading indications. Calculating convergent and discriminant validities was used to measure validity. Table 7 shows how three measurements – alpha, Cronbach’s Dijkstra-rho Henseler’s coefficients, and composite reliability – have a cutoff value of 0.7. The average variance extracted (AVE) for each concept is more than 0.5, demonstrating the third convergent validity. The measurement model satisfies the requirements, as shown in Table 8. The three constructs’ and the entire questionnaire’s Cronbach’s alpha values were all higher than the suggested cutoff point of 0.7^21^ and ranged from 0.686 to 0.934, indicating good internal consistency. Additionally, every item’s factor loading value exceeded the acceptable level of 0.5.

##### Composite Reliability (CR)

The purpose of the reliability test is to determine how much measuring equipment can be depended on or trusted. The reliability of the indicators in this study was evaluated using the PLS approach. The composite reliability value had to be greater than 0.7 or a threshold value of 0.7 or above.^5,20,24^ Table 8 shows that in the research model, each variable had a composite reliability range value of 0.737–0.943, which was more than 0.70. It was determined from these findings that the research model met the composite reliability value and that this study could be considered dependable.

Average Variance Extracted (AVE) (AVE): The average variance retrieved was used to assess convergent validity.^25^ The threshold value of AVE≥0.50 for each contract is.^19–21,26^ In this study, Table 8 shows that the value range of AVE was 0.495 to 0.601. This means that the AVE value prevailed within the threshold value and well supported the model.

##### Cronbach’s Alpha (CA)

As indicated in Table 8, the current study met the suggested range for Cronbach’s alpha values, which is between 0.70 and 0.90.^6,21^ Cronbach’s alpha is a method for estimating the dependability of a single test delivery. Cronbach’s alpha is the mean of the reliability coefficients derived by splitting a test in half for all possible item combinations. Cronbach’s alpha reliability coefficient typically falls between 0 and 1. George and Mallery’s^38^ research offers the following guidelines: A score of at least 0.9 is considered great, 0.8 is considered good, 0.7 is considered acceptable, 0.6 is considered doubtful, 0.5 is considered bad, and 0.5 is considered unsatisfactory. It is important to note that the value of growing the number of items in the scale has decreasing returns, although raising the value of alpha is somewhat reliant upon the number of items in the scale. An alpha of 0.8 is perhaps an acceptable objective to go toward, which is something else that should be mentioned. It should be noted that a high Cronbach’s alpha score indicates a high level of internal consistency.

#### 3.4.2 Discriminant Validity

One aims for the Cross-Loading value to be larger than 0.70 and the AVE value of each variable to have a value that is greater than 0.5 while undertaking discriminant validity testing. This ensures that each notion of each latent variable is distinct from the concepts of the other variables.^5^ According to the findings, each of the five latent variables had cross loading values that were greater than 0.70 and AVE values that were greater than 0.5. In other words, the results of the discriminant validity test indicate that all of the variables that were utilized in the study may be regarded as excellent or valid. The discriminant validity was examined in two stages: the first stage used the Fornell-Larcker criteria, and the second stage used the Heterotrait-monotrait ratio (HTMT). The following are two indices that will be discussed:

##### Fornell-Larcker Criterion

Following the recommendation of Fornel and Larcker^42^, We computed AVE’s square root. Table 9 shows that the discriminant validity is acceptable since the square root of AVE (provided in the diagonal of the correlation matrix) of each construct is larger than the correlation coefficients of any construct with other constructs.

**Table 9.** Fornell-Larcker Criterion.

	Attitude	Knowledge	Practices
Attitude	0.667		
Knowledge	0.469	0.521	
Practices	0.730	0.724	0.597

According to Fornell and Larcker (1981), the correlations between the latent variables should be smaller than the square root of the AVE of each latent variable.

##### Heterotrait-Monotrait Ratio (HTMT)

The HTMT results, which showed that all values were significantly different from 1, and the HTMT ratio of correlation in Table 10, which demonstrates that all values are below the cutoff of 0.90, both proved the discriminant validity of the reflective constructs. Table 10 shows the discriminant validity results using the HTMT correlation ratio. All constructs are discriminately valid since the confidence interval does not include a zero value. This implies that each variable is distinct from the others. The HTMT was estimated using the Smart PLS3 program, and it was discovered that all HTMT values were below the threshold value (HTMT<1). [Hair et al, 2014 6, 26–28] The HTMT ratio of correlation in Table 10 reveals that all values are less than 0.90, demonstrating the discriminatory validity of the reflective constructs. The measurement model’s data evaluations show that the construct is reliable and valid.

**Table 10.** Heterotrait-Monotrait Ratio (HTMT).

	Attitudes	Knowledge	Practices
Attitudes			
Knowledge	0.424		
Practices	0.860	0.700	

##### Multicollinearity Issue of Structural Model

Multicollinearity is a difficulty for the structural model. The VIF is a way to measure the overlap between the predictor variables in a multiple regression. This is done when evaluating SEM to ensure that the overlap problem has been fixed. When the VIF is greater than 5. This means that there may be a collinearity problem between the dimensions.^5,20,26,29^ Given that the VIF value of the SEM is less than a threshold value (VIF<5), which is between 1.031 and 4.595, there is no collinearity among the latent variables in this study.

##### Coefficient of Determination (R^2^)

R^2^ is the coefficient of determination, which measures the total variation in the dependent variable caused by the independent variables. When the value R is close to 0, it means the coefficients are insignificant. The value lies in the range of 0–1. When it is closer to 1, it shows a high significance of coefficients. . R^2^ value describes the internal variability of the proposed model.^5,20^ In general, an *R*^2^ of 0.10 is significant.^19^ However, an *R*^2^ value of 0.60 is considered significant in PLS-SEM, 0.33 is considered moderate, and 0.19 is considered weak.^25^

The R^2^ value of the practices to adopt hybrid rice 68% variability of the model The remaining 31.6%, however, can be explained by variables not considered in the research model. The attitude variable has an R^2^ value of 0.150, indicating that farmers’ attitudes may account for 15% of hybrid rice adoption, with the remaining 85% explained by factors not included in the research model.

#### 3.4.3 Calculation of the Goodness-of-Fit Index

The fit model is not the focus of structural equation modeling using partial least squares. According to Tenenhaus et al.^30^ and Abid et al.,^31^ the GoF is a method for globally validating a PLS route model. A good fit model shows that a model is reasonable and believable. Heseler and colleagues [.^32^) The average community (AVE values) and average R^2^ value are used in the calculation(s). The value of GOF = 0.642 shows that the fit index is good enough to verify the validity of the global model using the method published by Tenenhaus et al [.^30^) The model’s goodness of fit is determined using the Eq(i) as follows (2005):

$$GOF=\sqrt{AVE\times R^{2}}$$

The viability of a structural equation model was thoroughly evaluated by calculating the goodness of fit (GoF) value using the following method [,^30^in.^16^]The GoF of the model was determined to be 0.813 using this formula. This demonstrates the acceptability and interpretability of the model.

##### Standardized Root Mean Square Residual (SRMR)

SRMR is a measure of the mean absolute value of the correlation residuals. The study model has a decent match when SRMR<0.08; nevertheless, a lower SRMR is deemed to be a superior fit. Table 11 shows that the conceptual model’s SRMR was 0.134, indicating that the conceptual model matched well. We verified that the model’s chi-square fitness was 1917.835. Other model fit indices, such as the normed fit index (NFI = 0.411), are similar. The NFI is very sample size dependent^43–51^. NFI is therefore no longer used to assess model fit [Bentler 1990;.^18^]According to a thorough evaluation of both the internal (structural) and external (measurement) models. All of the hypotheses were confirmed after being determined to be statistically significant.

**Table 11.** R Square.

	R Square	R Square Adjusted
Attitudes	0.157	0.150
Practices	0.689	0.684

**Table 12.** Goodness-of-fit index summary.

	Saturated Model	Estimated Model
SRMR	0.134	0.134
d_ULS	7.769	7.769
d_G	4.052	4.052
Chi-Square	1917.835	1917.835
NFI	0.411	0.411

Note*: SRMR = Standardized root mean square residual, d_ULS = squared Euclidean distance, d_G = geodesic distance, and NFI = Normed fit Index*

##### Normed Fit Index (NFI)

The suggested model’s chi-square value is calculated using the Bentler-Bonett index (NFI), an incremental fit measure that compares it to a valuable benchmark to assess how well something fits.^33^ NFI values above 0.9 are acceptable for factor models since they demonstrate a good match in the Table 12.^34^ For composite models, the NFI levels have not yet been determined. Since the NFI does not penalize growing parameter values, it should be utilized cautiously when comparing models.

##### D_ULS and D_G

According to Heenseler et al. [,^35^) there are two methods for calculating this disparity: dLS and dG. The model becomes more accurate as the dULS decreases. The model used in this experiment is more appropriate, as shown by the geodesic (dG) inconsistency of 0.516.

## 4. Discussion

The overall effect of adopting hybrid rice had a high path coefficient (β = 0.645) with how people felt about factors related to how it would be used. Knowledge-related factors were found to be the second most important factor (β = 0.396) in the overall medium that affected the adoption of hybrid rice, and knowledge had a direct effect (β = 0.326) on the adoption of hybrid rice in Bangladesh. Figure 5 and Table 7 indicate the direction and breadth of links in farmers’ knowledge and attitude, as well as their direct consequences on the practices model. Farmers’ knowledge has a positive and significant link with hybrid rice practices (β_1_ = 0.326, p < .05); hence, null hypothesis 1 is accepted. The results conclusively demonstrate that farmers’ knowledge directly influences their adoption of hybrid rice. Based on the model’s size (i.e., β_1_ = 0.326, p < .05) and direction (i.e., positive association), hybrid rice adoption practices may rise by 0.32 units for every unit gain in knowledge. The second hypothesis (H_2_) postulates that attitude directly affects hybrid rice adoption practices. *H_2_* is supported, as the SEM demonstrates a positive and significant relationship between the two variables (β_2_ = 0.645, p < .05). There is a significant relationship between farmers’ knowledge and attitude in this study (β3 = 0.396, p < .05); thus, H_3_ is accepted. This means that hybrid rice adoption behavior was directly affected by farmers’ attitudes and indirectly affected by farmers’ knowledge (via attitudes). The results suggest an insignificant impact of farmers’ knowledge, attitudes and practices on the adoption level of hybrid rice by rice farmers in the Sherepur district of Bangladesh as well as developing countries in the world. This document provides the most recent and thorough overview of the PLS-SEM approach and the metrics used to evaluate its answers. While rice is the most widely consumed food in the world, the results of PLS-SEM are extremely important in providing farmers with suggestions for hybrid rice varieties that can adapt to rice-producing locations by raising farmers’ KAPs. Thus, the hybrid rice technology adoption campaign intends to achieve food security and sufficiency via continued engagement with national and local governments, commercial sectors, and farmers as they start on a substantial rice productivity effort.

## 5. Conclusions and Recommendations

The quantity of knowledge among farmers has a significant effect on the degree of satisfaction with hybrid rice adoption. Farmers’ perceptions have a considerable impact on the adoption of hybrid rice in the district of Bangladesh and farmers’ behaviors have a significant influence on the adoption of hybrid rice in Bangladesh. A knowledge index of sample farmers revealed that, compared to nonadopters, the majority of adopters have sufficient information regarding hybrid rice. According to the attitude index, the majority of adopters had medium to high attitudes. These findings demonstrated that adopters had a more favorable opinion toward hybrid rice than nonadopters. As a result, by incorporating labor-saving technologies into extension programs, extension officers would have access to the information they need to promote the widespread adoption of hybrid rice. Raising farmer awareness and understanding about the value of hybrid rice is an important step toward increasing the demand for hybrid rice. In addition, because of cheaper costs, agricultural producers may have a competitive edge in delivering to local communities. Increased farmer KAPs and the development of a hybrid rice quality certification system are required to improve this semiformal economy. Additional efforts by the government, for-profit businesses, and NGOs are also needed.

## 6. Limitations of the Study

We must acknowledge several limitations of this research. First, very few sample sizes were considered for this study, the results of which do not display a whole picture of the country. The second drawback of this study based on cross-sectional data, which means that the information was acquired during a limited time period and might not be representative of the population as a whole. Third, there are certain restrictions associated with the PLS-SEM soft modeling technique that was utilized in this research. Although this method does not need normalcy, the lack of a global goodness-of-fit index is a significant limitation.^36–38^

## Data Availability

The corresponding author can provide the data described in this study upon request.
